# ISUP Grade Prediction of Prostate Nodules on T2WI Acquisitions Using Clinical Features, Textural Parameters and Machine Learning-Based Algorithms

**DOI:** 10.3390/cancers17122035

**Published:** 2025-06-18

**Authors:** Teodora Telecan, Alexandra Chiorean, Roxana Sipos-Lascu, Cosmin Caraiani, Bianca Boca, Raluca Maria Hendea, Teodor Buliga, Iulia Andras, Nicolae Crisan, Monica Lupsor-Platon

**Affiliations:** 1 Department of Anatomy and Embryology, “Iuliu Hatieganu” University of Medicine and Pharmacy, 400012 Cluj-Napoca, Romania; t.telecan@gmail.com; 2 Department of Pathology, Country Emergency Clinical Hospital, 400347 Cluj-Napoca, Romania; 3 Department of Computer Science, Faculty of Mathematics and Computer Science, “Babes-Bolyai” University, 400157 Cluj-Napoca, Romania; roxana.sipos1@ubbcluj.ro; 4 Department of Medical Imaging, “Iuliu Hatieganu” University of Medicine and Pharmacy, 400012 Cluj-Napoca, Romania; ccaraiani@elearn.umfcluj.ro (C.C.); petresc.bianca@elearn.umfcluj.ro (B.B.); monica.lupsor@umfcluj.ro (M.L.-P.); 5 Department of Radiology, “George Emil Palade” University of Medicine, Pharmacy, Science and Technology”, 500139 Târgu Mureș, Romania; 6 Department of Pathology, “Iuliu Hatieganu” University of Medicine and Pharmacy, 400012 Cluj-Napoca, Romania; maria.bungardean@elearn.umfcluj.ro; 7 Department of Cardiology, Heart Institute Niculae Stăncioiu, 400001 Cluj-Napoca, Romania; teodor.cons.buliga@elearn.umfcluj.ro; 8 Department of Urology, “Iuliu Hatieganu” University of Medicine and Pharmacy, 400012 Cluj-Napoca, Romania; dr.iuliaandras@elearn.umfcluj.ro (I.A.); drnicolaecrisan@elearn.umfcluj.ro (N.C.); 9 Department of Urology, Clinical Municipal Hospital, 400139 Cluj-Napoca, Romania; 10 Department of Medical Imaging, Regional Institute of Gastroenterology and Hepatology “Prof. Dr. Octavian Fodor”, 400162 Cluj-Napoca, Romania

**Keywords:** clinically significant prostate cancer, textural analysis, artificial intelligence, machine learning, multi-parametric magnetic resonance imaging

## Abstract

**Background:** Prostate cancer (PCa) represents a matter at the forefront of healthcare, being divided into clinically significant (csPCa) and indolent PCa based on prognostic and treatment options. Although multi-parametric magnetic resonance imaging (mpMRI) has enabled significant advances, it cannot differentiate between the aforementioned categories; therefore, in order to render the initial diagnosis, invasive procedures such as transrectal prostate biopsy are still necessary. In response to these challenges, artificial intelligence (AI)-based algorithms combined with radiomics features offer the possibility of creating a textural pixel pattern-based surrogate, which has the potential of correlating the medical imagery with the pathological report in a one-to-one manner. **Objective**: The aim of the present study was to develop a machine learning model that can differentiate indolent from csPCa lesions, as well as individually classifying each nodule into corresponding ISUP grades prior to prostate biopsy, using textural features derived from mpMRI T2WI acquisitions. **Materials and Methods:** The study was conducted in 154 patients and 201 individual prostatic lesions. All cases were scanned using the same 1.5 Tesla mpMRI machine, employing a standard protocol. Each nodule was manually delineated using the 3D Slicer platform (version 5.2.2) and textural parameters were derived using the PyRadiomics database (version 3.1.0). We compared three machine learning classification models (Random Forest, Support Vector Machine, and Logistic Regression) in full, partial and no correlation settings, in order to differentiate between indolent and csPCa, as well as between ISUP 2 and ISUP 3 lesions. **Results**: The median age was 65 years (IQR: 61–69), the mean PSA value was 10.27 ng/mL, and 76.61% of the segmented lesions had a PI-RADS score of 4 or higher. Overall, the highest performance was registered for the Random Forest model in the partial correlation setting, differentiating between indolent and csPCa and between ISUP 2 versus ISUP 3 lesions, with accuracies of 88.13% and 82.5%, respectively. When the models were trained on combined clinical data and radiomic signatures, these accuracies increased to 91.11% and 91.39%, respectively. **Conclusions**: We developed a machine learning decision support tool that accurately predicts the ISUP grade prior to prostate biopsy, based on the textural features extracted from T2 MRI acquisitions.

## Simple Summary

Prostate cancer is the most frequent and impactful malignancy in male patients. Although multi-parametric magnetic resonance imaging (mpMRI) of the prostate has enabled notable progress in terms of image resolution and prostate cancer detection, it does not hold a one-to-one correspondence with the pathological report; thus, a prostate biopsy is still needed in order to confirm the suspicion of malignancy. Recent advancements in textural analysis and machine learning (ML) classification models have attempted to create non-invasive diagnostic surrogates, bypassing the need for confirmatory biopsy. Starting from this hypothesis, we aimed to create an ML-based classification algorithm that can accurately predict the histology of prostate nodules, based on their textural characteristics derived from the mpMRI scans and integrated clinical data.

## 1. Introduction

Prostate cancer (PCa) represents a matter at the forefront of healthcare, as it comprises 29% of newly detected all-site malignancies and 11% of cancer-related deaths in the male population annually [1]. The International Society of Urological Pathology (ISUP) classifies PCa into five categories, based on their aggressiveness, from ISUP 1 (least aggressive) to ISUP 5 (most aggressive) [2]. According to the European Association of Urology (EAU), PCa can be further divided into two main categories: clinically insignificant—ciPCa (ISUP 1) and significant—csPCa (ISUP of 2 or higher) [3]. This dichotomy reflects the difference in terms of progression rates and treatment strategies for the two categories: ciPCa registers progression to csPCa in 2.91% of cases over the course of 8 years, thus being suitable for active surveillance (AS) strategies [4], whereas csPCa may progress in 12% of cases within 6 to 12 months from the initial diagnosis, leading to the need to employ active treatment strategies, such as radical prostatectomy or curative radiation therapy [5]. Additionally, regarding csPCa, the threshold above which adjuvant radiation or androgen deprivation therapy are needed is considered as being ISUP 3 or above [6].

The current guidelines [7] suggest that any patient with clinical (positive digital rectal examination) or biochemical suspicion (prostate specific antigen over 4 ng/mL) of PCa should undergo multi-parametric magnetic resonance imaging of the prostate (mpMRI). Based on the Prostate Imaging Reporting and Data System (PI-RADS) criteria [8], every lesion classified as PI-RADS 3 or higher must be sampled through a prostate biopsy, in order to rule out or confirm the presence of a possible malignant nodule within the gland. However, the radiologist’s interpretation of the mpMRI scans is often subjective, with the overall interobserver agreement reaching 76.5% [9], while dropping to 43% for equivocal PI-RADS 3 lesions [10]. Moreover, the diagnostic capability of the biopsy procedure is limited to the sampled core. Compared to the radical prostatectomy specimen, prostate biopsy falsely classifies patients in the ciPCa category in 25.5% of cases [11], partially due to nodule targeting and tangential sampling.

As a response to these diagnostic challenges, clinical and paraclinical biopsy alternatives have been developed in order to obtain an accurate, non-invasive histopathological surrogate. Palmisano et al. [12] elaborated a PSA and PSA ratio-based nomogram, which was able to predict the presence of csPCa in PI-RADS 3 nodules with a sensitivity and specificity of 70% and 80%, respectively. Likewise, in terms of radiological innovations, Lambin et al. [13] developed and introduced the subdomain of radiomics for the first time in 2012. Defined as a mathematical approach to medical imaging, radiomics extracts information about the spatial arrangement of pixels, their pattern, and gray-scale intensities in a quantifiable and reproducible way [14]. Through the integration of these features into artificial intelligence (AI)-based algorithms, visually appreciable features such as the shape or texture of a given region of interest can be correlated with the in vivo heterogeneity and malignancy potential of various tissues, serving as additional decision support tools in rendering a diagnosis or performing an organ biopsy [15].

Considering the large amount of data that prostate mpMRIs harbor, it has been suggested that the most suitable algorithm architecture to be fully integrated in the routine diagnostic workflow would be a machine learning (ML) model, trained with textural features mined from a limited number of the mpMRI acquisitions, either with high contrast of the targeted structures or good anatomical landmark identification [16]. Due to the fact that T2-weighted images (T2WIs) are most commonly used by clinicians when performing MRI-guided prostate biopsies, ML algorithms trained with textural features extracted from T2WI sequences have the best chance for adoption in day-to-day clinical practice at a larger scale. Existing studies with similar design have supported the feasibility of this concept, reporting accuracies of 89% and 78% in terms of differentiating benign from malignant nodules and clinically insignificant from csPCa lesions, respectively [17].

The primary aim of the present study is to develop a machine learning model that can classify and differentiate indolent from csPCa lesions prior to prostate biopsy, using textural features derived from mpMRI T2WI acquisitions. The secondary objectives focused on integrating parameters such as PSA, age, and digital rectal examination into the ML architecture, in order to assess the accuracy of a combined clinical and radiomics-based algorithm.

## 2. Materials and Methods

### 2.1. Patient and Clinical Data Selection

The present study included all patients diagnosed with prostate adenocarcinoma via MRI–transrectal ultrasound (MRI-TRUS) fusion prostate biopsy in our department between June of 2022 and June of 2024. The indication of prostate biopsy was established based upon positive digital rectal examination and/or total serum prostate specific antigen (PSA) values over 4 ng/mL, in addition to a prostate mpMRI scan confirming the presence of at least one suspicious lesion graded PI-RADS 3 or higher [18]. All serum PSA determinations were made using Elecsys^®^ Total PSA Electrochemiluminescence Immunoassay (ECLIA) kits and the Cobas^®^ e 601 Automated Analyzer platform (Roche Diagnostics, Basel, Switzerland). Patients who presented prostate biopsy or acute prostatitis in the previous 12 months, or transurethral resection of the prostate at any given moment in their past medical history, were excluded, due to anatomical and textural changes of the gland.

Collected data of interest included the following: age, digital rectal examination assessment, total PSA value, PI-RADS score and size of each individual lesion, prostate volume, biopsy approach, total number of sampled biopsy cores and number of targeted cores per lesion, as well as the global and per-lesion ISUP grades. All data were gathered in a prospective manner.

The protocol was elaborated in accordance with the Declaration of Helsinki regarding the Ethical Principles for Medical Research Involving Human Patients, which was approved by the local Ethics Committee (No. 245/09.07.2022, issued by the University of Medicine and Pharmacy “Iuliu Hațieganu”, Cluj-Napoca, Romania). Individual patient consent was obtained prior to study enrollment.

### 2.2. Pre-Biopsy mpMRI Acquisition Protocol

All examinations were performed in a single institution, using the same 1.5 Tesla MRI scanner (MAGNETOM Aera^TM^, Siemens Healthcare, Erlangen, Germany) with a 16-channel phased-array body coil. All patients were examined using the same scanning protocol, which included T2-weighted turbo spin-echo (TSE), high-resolution sequences in three orthogonal planes (coronal, sagittal, and oblique–axial), as well as T1-weighted TSE acquisition in the axial plane. Diffusion-weighted images (DWIs) were obtained for the axial sequences at b values of 50, 400, 800, 1000, and 1500. Apparent diffusion coefficient (ADC) maps were automatically rendered via the scanner’s software, based on DWI acquisitions. The last step comprised dynamic contrast-enhanced (DCE) acquisitions, rendered following free-hand administration of gadobutrol (Gadovist^TM^ 1.0; Bayer Schering Pharma AG, Berlin, Germany; dose of 0.1 mmol/kg). Each sequence was set at 25–35 slices, each slice having a thickness of 3–4 mm. The field of view was set at a 200–220 mm width, with a matrix size of 256 × 320.

All mpMRIs were interpreted by two radiologists, who reached a consensus in terms of the diagnosis, location, and individually attributed PI-RADS score of the lesion(s).

### 2.3. Oblique-Axial T2WI Segmentation

**Figure 1:**
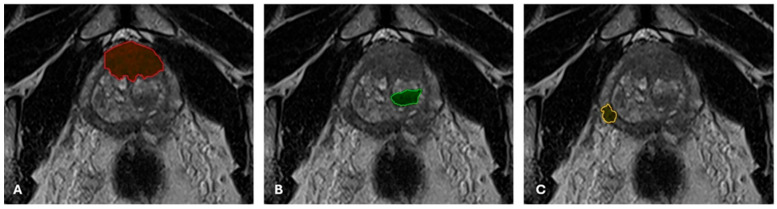
Individual segmentation of a case with multiple nodules, each area being analyzed as a stand-alone dataset. (**A**). PI-RADS 5 lesion, bilaterally located in the anterior fibromuscular stroma (red area). (**B**). PI-RADS 4 lesion, located in the left transitional zone in the middle segment of the gland (green area). (**C**). PI-RADS 4 lesion, located in the peripheral zone of the middle segment of the right prostatic lobe (yellow area).

The T2WI TSE oblique–axial acquisitions of each patient were anonymized and exported for subsequent segmentation using the 3D Slicer open-source software (version 5.2.2) [19]. Each nodule was manually delineated on each consecutive slice and marked as individual regions of interest (Figure 1).

Prior to textural feature extraction, all acquisitions were normalized to a gray-level range of 0–256 using the “SimpleScaleImageFilter” function. In order to create isotopic voxels, we used the “bspline” interpolation algorithm, resampling the gap of approximately 4 mm between two consecutive slices to 0.3 mm.

**Figure 2:**
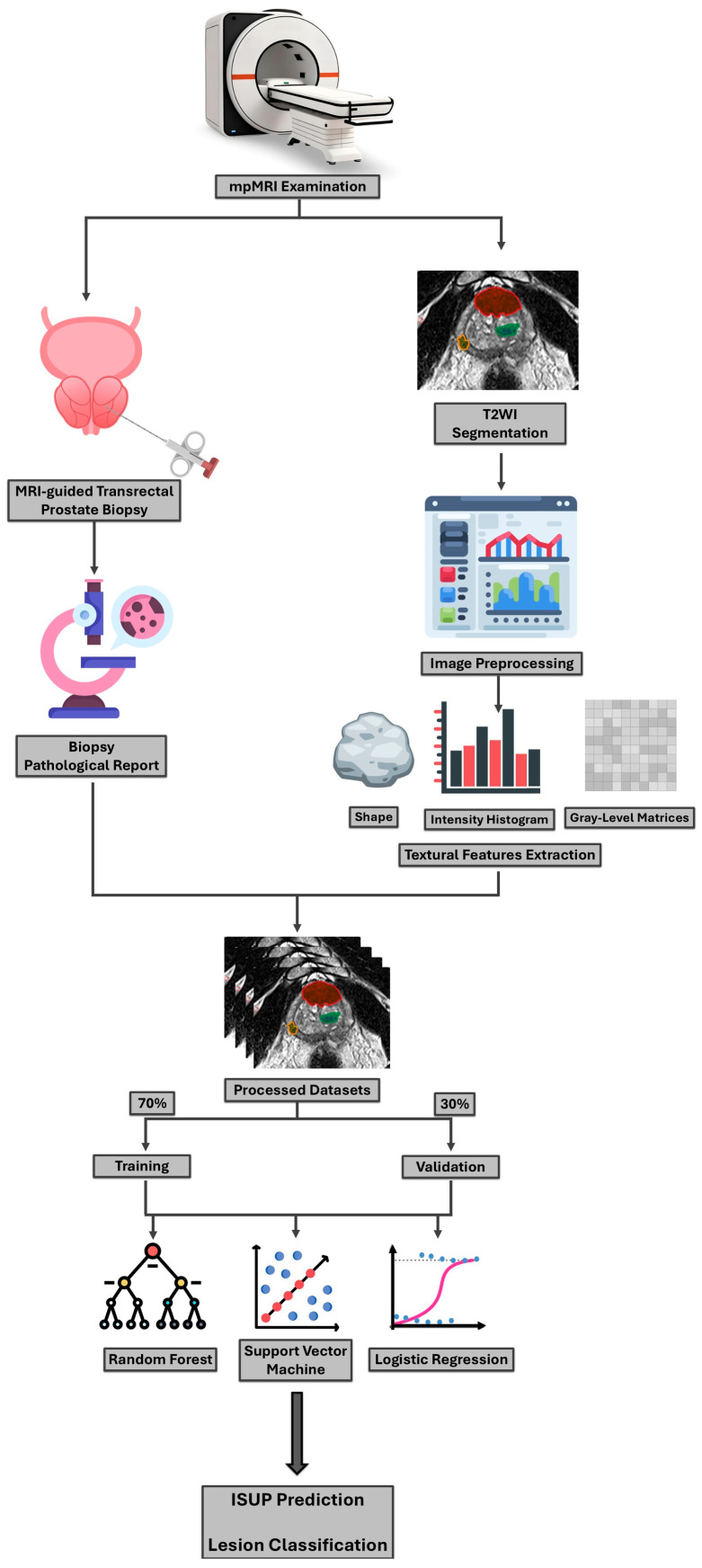
Graphical description of the study protocol.

The processed segmentations were used to extract the characteristic textural features for each ISUP grade, which were further employed in the training phase of the machine learning model. In summary, the study protocol and step-by-step workflow are graphically represented in Figure 2.

### 2.4. MRI-TRUS Fusion Biopsy

All patients underwent transrectal MRI-US fusion biopsy, performed using the Hitachi-Aloka Arietta 70a platform (Hitachi Ltd., Chiyoda, Tokyo, Japan), with real-time virtual sonography (RVS) software and a rigid registration system. The used endocavitary probe was the C41V “end-fire” transducer, with a 2–10 MHz frequency range and a 200-degree field of view scan angle. The biopsies were carried out in an outpatient setting and under local anesthesia, by a senior urologist with 8 years of experience, using the Bard Magnum^TM^ reusable core biopsy gun (Becton, Dickinson and Company, Franklin Lakes, NJ, USA) with disposable 18-gauge biopsy needles. The marked nodules were sampled with 2–4 targeted cores per lesion, followed by standard double-sextant systematic biopsy.

### 2.5. Pathology

All biopsies were assessed by the same senior pathologist, with over 15 years of experience in the uro-oncological field. The total and individual lengths of the cores were measured, and the samples were processed and entirely embedded in paraffin blocks. The histological diagnosis was established based on all tissue slides, sectioned at a thickness of 2–3 μm and colored with standard hematoxylin–eosin stain. Immunohistochemical markers such as Alpha-methylacyl-CoA-racemase (AMACR), Anticytokeratin antibody 34 beta E12 (CK34betaE12) and tumor protein 63 (p63) were used whenever the specialist considered it necessary. The ISUP grade was calculated globally after assessing the Gleason Score for each positive biopsy, according to the Guidelines of the International Society of Urological Pathology [20].

### 2.6. Software Development

The ML algorithm was developed on a laptop with the following characteristics:

- Operating system: Windows 10 (Microsoft Corporation, Redmond, WA, USA);
- Processor: 12th Gen Intel^®^ Core™ i7-10610U (Intel Corporation, Santa Clara, CA, USA);
- Central processing unit (CPU): 1.80 GHz;
- Random-Access Memory (RAM): 16 GB;
- Graphics Processing Unit (GPU): Intel^®^ UHD Graphics;
- System type: 64-bit operating system on an x64-based processor.

The manual 2D per-nodule segmentations were converted into the NRRD (Nearly Raw Raster Data; .nrrd) format. For each lesion, both overall renderings and individual slices were analyzed. Prior to extraction of textural features, we performed discretization of the datasets. In order to reduce the image intensity and minimize the impact of noise, the intensity range was split into 32 bins, with each bin corresponding to 8 distinct gray levels.

To extract the textural features of each delineated region of interest, we used the PyRadiomics library (version 3.1.0) [21], which we employed to obtain the first-order features for the classification algorithm, extracted from the gray-level histogram and shape, as well as second-order features, such as the gray-level co-occurrence matrix (GLCM), gray-level dependence matrix (GLDM), gray-level size zone matrix (GLSZM), gray-level run length matrix (GLRLM), and neighboring gray-tone difference matrix (NGTDM) [22,23,24]. Consistent with our previously published protocols [25,26], 107 textural parameters were extracted per area of interest, which were eventually converted into TSV (Tab-Separated Values; .tsv) format.

Next, each classification task was subdivided into three models:

- Clinical: based on features such as age, digital rectal examination findings, PSA density, PI-RADS score, and the lesion’s maximum diameter;
- Radiomic: based on textural features only;
- Combined: an integrative model that combines clinical data with radiomic features.

To build each classification model (clinical, radiomic, and combined radiomic–clinical), we incorporated three machine learning algorithms: Random Forest (RF), Support Vector Machine (SVM), and Logistic Regression (LR). All models were calibrated using the associated implementations in scikit-learn (version 1.5.2). The main characteristics are presented in Table 1. Hyperparameter tuning was conducted using Random Search, allowing for the efficient exploration of a diverse range of hyperparameter combinations given the size of the developed dataset (201 cases, 107 variables). Through this process, we determined that 20 trees were sufficient for our classification task, as increasing the number of trees showed diminishing returns in accuracy, while significantly increasing the training time. Finally, we divided the data into training and testing groups using a 70% to 30% ratio, respectively. For the separate per-ISUP grade analysis, data augmentation was applied for the ISUP 3 cases, which were oversampled twice by employing the random oversampling method.

### 2.7. Statistical Analysis

Hyperparameter tuning using Random Search was performed before textural feature selection, ensuring that the chosen model configurations were optimal across different feature subsets. The impact of feature reduction on model performance was then evaluated using the three correlation-based approaches, starting from the main correlation matrix.

Initially, for the radiomics-only model, all machine learning classifiers were trained and tested on all 107 features and clinical parameters, using the “Full correlation” setting, which attributed a complete linear relationship to each variable. The second analysis was based on the “No correlation” principle, leaving only 93 out of 107 parameters to be further computed, alongside with the clinical parameters for the combined models. These features are illustrated in Supplementary Table S1. Finally, the third adjustment was made following the “Partial correlation” principle, where the parameters were examined based on their corresponding clusters. This strategy narrowed the number of included textural parameters into the radiomics-only model to 28 (Supplementary Table S1), which were further combined with the chosen clinical features.

The feature importance selection was conducted in a two-step approach. First, we used the aforementioned correlation matrices, with each of the 107 features being tested against the preset correlation coefficient. For the “Full” and “Partial correlation” settings, the coefficient thresholds were 0.85 and 0.65, respectively, and features with higher correlation indices were excluded. Second, the Random Forest model used the Mean Decrease of Impurity (MDI) as an additional default metric to measure feature importance; meanwhile, for the Logistic Regression and Support Vector Machine models, the Mean Decrease of Accuracy (MDA) was used.

For each classification model and correlation degree, the following statistical parameters were calculated: true positive and negative rate (TPR and TNR), positive and negative predictive value (PPV and NVP), false positive and negative rate (FPR and FNR), false discovery rate (FDR), and accuracy (Supplementary Figures S1 and S2). Additionally, each subset was statistically analyzed using Student’s *t*-test. A *p*-value below 0.05 was considered indicative of statistical significance.

## 3. Results

### 3.1. General Characteristics of the Study Group

The study included 154 patients with a total of 201 lesions. The general characteristics of the cohort are provided in Table 2. The median age of the study group was 65 years and the mean PSA value was 10.27 ng/mL, ranging from 3.5 ng/mL to 70 ng/mL with a standard deviation of 9.06, thus demonstrating a wide distribution of values around the average. A total of 76.61% of the segmented lesions had a PI-RADS score of 4 or higher.

### 3.2. Performance of the Trained Algorithms for Differentiating ISUP 1 from ISUP 2–5 Lesions

Each machine learning model was tested under the full, no, and partial correlation settings, and the results are presented in Table 3 and Table 4, and Supplementary Table S2.

**Figure 3:**
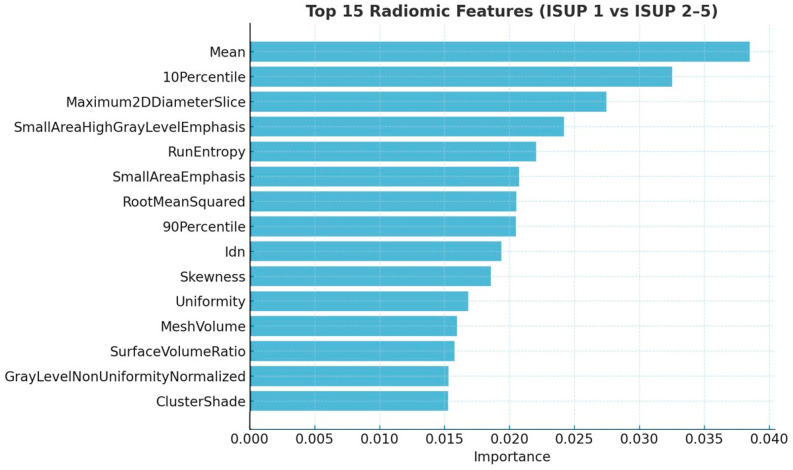
Top 15 radiomic features extracted through Mean Decrease of Impurity function for the ISUP 1 versus ISUP 2–5 classification, used to train the Random Forest model. Idn = Inverse Difference Normalized.

The top 15 features for distinguishing ISUP 1 from higher-grade lesions are shown in Figure 3. To assess the robustness of these features under dimensionality reduction, we compared rankings across the filtered feature sets. Ten out of the top fifteen features from the full model were retained in the 93-feature set, and ten also appeared in the 28-feature set. Notably, eight features were common across all three rankings, underscoring their stability and relevance. These were Mean, 10Percentile, RootMeanSquared, Uniformity, SurfaceVolumeRatio, SmallAreaHighGrayLevelEmphasis, GrayLevelNonUniformityNormalized, and SmallAreaEmphasis.

Overall, we determined that the best result was derived from the Random Forest classification model employing a partial correlation approach. For the radiomic model, it yielded the highest true positive rate and positive predictive value (93.75% and 84.28%, respectively), demonstrating a high probability of classifying correctly each case as indolent (ISUP 1) or clinically significant PCa (ISUP 2–5). Although SVM with full correlation presented the best specificity (92%), it provided a below-average negative predictive value (48.65%), thus making the Random Forest model in the partial correlation setting the overall most-specific model, presenting a true negative rate of 82.5% and a negative predictive value of 92.87%. Additionally, this model provided an accuracy of 88.13%, which indicated it as the most reliable one. Furthermore, when combining clinical and radiomic data, the Random Forest classifier reached an accuracy of 91.11% in the partial correlation matrices.

### 3.3. Performance of the Trained Algorithms for Differentiating ISUP 2 Versus ISUP 3 Lesions

To further assess the utility of the proposed classification models as diagnostic tools, we performed a sub-analysis of ISUP 2 versus ISUP 3 lesions alone, applying the same correlation thresholds (Table 5 and Table 6, and Supplementary Table S3).

**Figure 4:**
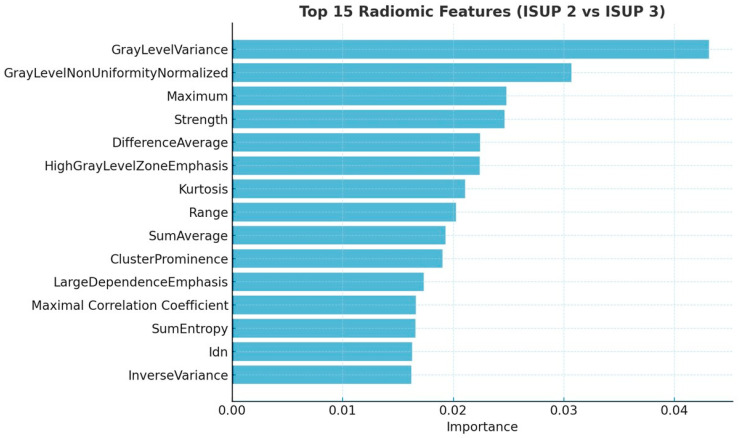
Top 15 radiomic features extracted through Mean Decrease of Impurity function for the ISUP 2 versus ISUP 3 classification, used to train the Random Forest model. Idn = Inverse Difference Normalized.

The classification between ISUP 2 and ISUP 3 lesions yielded a different set of dominant features, shown in Figure 4. These included GrayLevelVariance, Kurtosis, SumAverage, ClusterProminence, and Maximum, suggesting a stronger role for texture and intensity-based parameters in this more subtle differentiation task.

While the overall overlap was slightly lower, several key features remained stable across filtered feature sets. Six features were common to both the full and 93-feature model, while five features overlapped between the full and 28-feature model. Two features—GrayLevelVariance and GrayLevelNonUniformityNormalized—were consistently ranked among the top across all three models, highlighting their robust predictive value even under stricter dimensional constraints.

Similar to the previous classification strategy, the best sensitivity and accuracy were provided by the Random Forest model in the partial correlation setting, which correctly classified ISUP 2 and ISUP 3 lesions in 73.33% and 88% of cases for the radiomic model and 92.3% and 31.39% for the combined approach, respectively, while maintaining the lowest misclassification rates (26.67% and 7.69%) out of the three models. In contrast, the Support Vector Machine model was biased, identifying most cases as ISUP 2 and misclassifying ISUP 3 lesions in 46.67% of cases.

## 4. Discussion

Active surveillance in prostate cancer represents a pressing concern for healthcare systems worldwide as, at present, men diagnosed with indolent PCa are four times more likely to undergo active surveillance protocols than in 2010 [27]. The cost of current AS strategies can range from USD 6200 [28] to over USD 12,000 per year for each patient [28]. Although, at first glance, the cost-efficiency target seems to be reached when compared to primary radical treatment—at over USD 16,000 for radical prostatectomy or nearly USD 30,000 for radiation therapy [29]—the average AS enrollment period must be taken into consideration, which can vary between 5 and 10 years, eventually exceeding 15 years as the population ages and life expectancy increases [7]. Of the yearly AS expenses, up to 22% are represented by the repeat prostate biopsy—a cost that can increase to 38% after 10 years of enrollment [30]. Therefore, through the integration of an ML-based decision support tool that can accurately characterize the suspect nodules in a non-invasive way (thus mirroring the pathological analysis of biopsy specimens) into the routine surveillance workflow, the associated financial burden on healthcare systems can potentially be reduced significantly.

Most studies have focused on differentiating between classes of prostate cancer, and few have tackled the distinction between benign and malignant nodules. Winkel at el. [31] proposed a machine learning model that classifies PCa nodules from the adjacent non-tumoral tissue, employing solely DCE acquisitions of 402 cases and reaching an accuracy of 90.9%. However, as the authors later concluded, a major limitation of the study was that all nodules and non-malignant tissues were sampled from the peripheral zone. The foremost drawback in analyzing the malignant nodule versus the whole gland is represented by the heterogeneity of the transitional zone, which contains hyperplastic nodules and various degrees of chronic prostatitis, cystic degeneration, or calcifications, which may translate as potential PCa mimickers. In order for the classification model to properly differentiate all these entities, it would need to be exposed and trained to a wide range of benign variations of the aforementioned co-existing conditions. This would eventually lead to a large data volume and, thus, a deep learning classification model (e.g., a convolutional neural network) may be more appropriate. Our study focused on 154 patients and 201 individual nodules, which limited the training of benign variations of the transitional zone, especially considering our model’s machine learning architecture.

The distinction between clinically insignificant PCa and csPCa is of therapeutic importance, as the latter must be sanctioned through radical approaches within 3 months from the initial diagnosis [32]. However, the initial diagnosis can be delayed by the biopsy sampling technique, especially if the urologist targets the nodule tangentially. To date, it has been hypothesized that most nodules present concentric heterogeneity, with the highest Gleason Grade being found at the core of the nodule. Brisbane et al. [33] demonstrated this concept by sampling the center of the lesion and immediate adjacent area separately, thus proving that csPCa is located in the center of the lesion, while ciPCa surrounds its periphery circumferentially, in an area with a width of 5 to 16 mm. Therefore, biopsy cores sampled tangentially to the nodule area tend to be comprised of ISUP 1 adenocarcinoma alone, thus failing to provide an accurate description of the histology of the whole lesion, potentially inadvertently classifying the patient in a lower risk group. This hindrance can be ruled out by a pre-biopsy global texture analysis of the nodule, which can offer additional information about csPCa within the nodule.

Our main objective was to develop a machine leaning algorithm that can accurately classify indolent from csPCa in the pre-bioptic phase in a patient’s management, specifically through employing textural features extracted solely from T2WI mpMRI acquisitions. The best performance was registered when using the Random Forest model in the partial correlation setting, which obtained an accuracy of 88.13% and a misclassification rate (false negative rate) of 6.25%. From a clinical point of view, these findings illustrate the possibility of integrating machine learning predictions as a decision support tool for radiologists when assessing a prostate nodule; more importantly, this tool provides a reliable non-invasive surrogate to prostate biopsy in active surveillance protocols, potentially leading to increased patient compliance. Moreover, through combining relevant, easily accessible, and inexpensive clinical data with parameters extracted from T2WI acquisitions alone, the model reached a sensitivity (true positive rate), specificity (true negative rate), and accuracy of 94.14%, 88.08%, and 91.11%, respectively. This has the potential to optimize the use of healthcare resources, shortening a screening or follow-up mpMRI scan from 45 min to 8–9 min if only T2WI scans are performed [34]. Additionally, by specifically distinguishing ISUP 2 from ISUP 3 nodules with an overall accuracy of 91.39%, patients at higher risk have the possibility of being prioritized in order to shorten the timeframe between the moment of diagnosis and radical and/or adjuvant treatment.

In terms of the choice of algorithm architecture, our study focused on machine learning approaches. Unlike their deep learning counterparts, ML models require structured data and can deliver interpretable results even if applied on smaller volume datasets [35]. Although medical images are usually considered large, unstructured data, through manually segmenting the lesions of interest and filtering the T2WI acquisitions via gray-level normalization and interpolation, we reduced the overall computational workload and pre-processed the images in a way that better highlights the potential patterns within the nodules. Moreover, the manual delineation allowed labeling and the supervised training of the algorithm to be more easily performed, which is suitable given the size of the dataset. Additionally, we employed three ML classification models using the same samples of textural features. The best performance was obtained with the Random Forest model, while the least performant was the Logistic Regression model. This can be explained by the fact that Logistic Regression classifier performs best when trained on binary data presenting linear relationships, whereas the Random Forest can process more complex, yet structured data. Although the Support Vector Machine model registered similar precision and accuracy as the Random Forest model, the latter was found to be superior in all three tasks. This is potentially due to the dataset’s size and feature dimensionality, as we did not perform preliminary selection for the conducted experiments. It has been suggested that SVM models are best trained on manually selected data or derived features that synthetize their entire subclass—a method known as principal component analysis [35].

Regarding the correlation degree, partial correlation yielded the best results in all models, but especially when employed with the Random Forest model. The fact that the models were trained on second-order gray-level matrices as textural features played a pivotal role, as these datasets tend to exhibit high correlation due to the similarity of the spatial patterns derived from the regions of interest. A high degree of correlation (full correlation) led to overfitting, in which case ML models cannot generalize and extract key features of the given datasets and further use them to classify unlabeled data in the testing phase. To the contrary, a low degree of correlation (no correlation) leads to underfitting, causing the algorithm to overlook important relationships between definitive features. Therefore, the partial correlation approach achieved the best results due to the fact that it retained useful dependencies between major data points while also preventing redundancy, thus allowing the ML models to extract the main discriminative textural features of PCa nodules. Moreover, partial correlation paired best with the Random Forest classifier, as the latter is not impaired by multi-collinearity and, through randomly selecting partially correlated data for each decision tree split, it can eventually generalize definitive features from a textural pattern, thus allowing it to classify new data with high accuracy.

A fundamental part of elaborating the machine learning- and texture analysis-based decision support tool is the choice of mpMRI acquisitions and, furthermore, whether a single or multiple sequence should be used for feature extraction. The vast majority of literature has reported an average of two acquisitions as being simultaneously used—most commonly, T2WI and ADC or DWI—while some studies have also added DCE-derived features. Chaddad et al. [36] developed a machine learning algorithm that identified and distinguished clinically insignificant from csPCa, as well as ISUP 2 from ISUP 3 nodules with an accuracy of 83.4% and 72.7%, respectively. The authors used 41 features extracted from T2WI and ADC scans and used them to train a Random Forest classifier. in another study, authored by Zhang et al. [37], textural parameters were extracted from T2 and diffusion-weighted images, which were analyzed using the Least Absolute Shrinkage Logistic Regression model. The reported accuracies for differentiating indolent from aggressive PCa were 85.3% and 90.1% in the training and validation phases, respectively.

Regardless of the sequence association, as T2-weighted images are ubiquitous in radiomics studies, the question regarding whether T2WI can provide sufficient information to be used as a stand-alone acquisition mode in decision support tool development processes has arisen. T2WI is considered to be more anatomical, depicting accurately the scanned structures and important surrounding landmarks, thus being the acquisition mode of choice for MRI-guided prostate biopsy. Taking this into consideration, we trained and tested the machine learning classification algorithms solely on T2WI texture analysis features, in order to better quantify the possibility of integrating such a decision support tool into the routine workflow of MRI-guided prostate biopsies. To date, similar studies that focused exclusively on T2-weighted images have been reported. Gong et al. [38] published a study based on 489 patients, in which they used 1345 texture features extracted from T2WI acquisitions to differentiate between ISUP 1 and ISUP 2–5 lesions. The authors manually segmented the whole prostate and the machine learning algorithm was based on the logistic regression classifier, reaching accuracies of 71.2% and 64.5% in the training and testing phases, respectively. These results might be hindered by the segmentation method, as contouring the whole gland offers a wide array of features due to the heterogeneity of the transitional zone, coupled with the choice of classifier which, as mentioned above, performs best with binary, linear data. A more recent study, authored by Duenweg et al. [39], obtained an 89.9% accuracy for detecting PCa. Their research was conducted in 279 patients and employed 10 main textural parameters extracted from T2WI sequences, 9 first-order features and 1 gray-level matrix (GLSZM), utilizing a Random Forest classifier. Our study reported similar accuracy (88.13% in the testing and partial correlation setting) when referring to the same ML model. However, our protocol was based mostly on second-order gray-level matrix features, which are considered more complex due to the fact that they take into consideration not only the intensity of pixels but also their spatial relationships, thus representing a more accurate method for deriving textural patters from the region of interest and, subsequently, their attribution to a subclass of prostate cancer, therefore providing the potential to develop a more robust classifier.

Another concern that should be discussed is the use of both 1.5 and 3 Tesla scanners for PCa diagnosis and textural feature extraction. Although the PI-RADS v2.1 guidelines [8] advise the use of 3 Tesla MRI machines, recent meta-analyses have proved the non-inferiority of the 1.5 Tesla alternative in terms of diagnostic sensitivity and specificity (70.6% and 41.7% of 1.5 Tesla scanners versus 69.5% and 48.8% for 3 Tesla scanners, *p* = 0.89). The only advantages that the 3 Tesla machines seem to offer are lower noise artifact rates and better extracapsular effraction evaluation [40]. In terms of textural feature extraction, there is no consensus regarding the superiority of 3 Tesla scanners, as multiple systematic reviews have reported the use of 1.5 Tesla machines in 18% to 27.27% of the included papers [41,42,43]. One suitable explanation would be the inherent image processing prior to feature extraction, such as gray-level normalization and interpolation, which may attenuate the noise artefacts retained by some 1.5 Tesla acquisitions.

Finally, our protocol focused on differentiating between the csPCa subclasses—namely, between ISUP 2 and ISUP 3—reaching the highest precision and accuracy (82.5% each) when using the Random Forest model with the partial correlation setting. Likewise, Fehr et al. [44] performed the classification of 114 ISUP 2 and 26 ISUP 3 lesions using a Support Vector Machine algorithm. Additionally, they addressed the class imbalance problem by applying synthetic oversampling techniques and further grouping the lesions into those associated with the peripheral and transitional zones. The authors achieved an accuracy of 81% and 76% in the all-site and peripheral zone-exclusive analyses in the unenhanced, original cohort, and 64% and 61% when the study group was synthetically oversampled to 200 cases per category, respectively.

Taking the above into account, a particular advantage of our study protocol is the comparison of three machine learning models, trained solely on one MRI acquisition mode (T2WI) at different levels of feature correlation, which reached similar accuracy to more complex, multi-acquisition models published in the existing literature.

## 5. Limitations

The present study faces a few limitations. First, only proven cancerous nodules were taken into consideration for this study, without a preliminary experiment to distinguish between benign conditions and malignant lesions. While the peripheral zone is relatively uniform, the transitional zone is far more heterogeneous and textural parameters may vary significantly, often mimicking PCa lesions. Future studies with larger cohorts and more complex classification architectures are needed in order to achieve satisfactory accuracy in this regard. Second, we used oversampling strategies in order to balance the ISUP 3 category, as it was disproportionally represented in the study data. This has the potential to create synthetically achieved parameters which may affect the interpretability of the results, thus reducing the generalization ability of the algorithm. Finally, the protocol included data from the same MRI scanner, provided by a single tertiary care center. This may limit the external validity of the algorithm and result in lower detection accuracy; therefore, further multi-center and multi-scanner studies are necessary.

## 6. Conclusions

To conclude, we developed an artificial intelligence-based model that differentiated indolent from clinically significant prostate cancer with high accuracy, as well as ISUP 2 from ISUP 3 nodules. By focusing exclusively on T2-weighted acquisitions, the proposed decision support tool can be easily integrated into standard prostate cancer diagnostic protocols, assisting radiologists and urologists alike. However, future external testing and validation studies based on independent cohorts of patients and scans obtained with mpMRI machines from different vendor are needed in order to safely generalize the presented results.

## Figures and Tables

**Table 1:** Summary of the main settings applied in the three machine learning models.

	Random Forest		Support Vector Machine		Logistic Regression	
	Parameter	Value	Parameter	Value	Parameter	Value
**ISUP 1 vs. ISUP 2–5**	Number of trees:	20	Regularization:	0.0001	Regularization:	0.001
	Random State:	None	Kernel:	Poly	Maximum number of iterations:	100
	Maximum Depth:	100	Degree:	16	Solver:	Liblinear
	Minimum Sample Split:	10	Probability:	True	Random state:	None
	Minimum Sample Leaf:	5	Class weight:	Balanced	Class weight:	Balanced
	Maximum Features:	Log2				
	Bootstrap:	True				
	Maximum Samples:	0.7				
	Cost Complexity Pruning Alpha:	0.04				
	Class-weight:	Balanced				
**ISUP 2 vs. ISUP 3**	Number of trees:	40	Regularization:	0.0001	Regularization:	0.1
	Random State:	None	Kernel:	Poly	Maximum number of iterations:	100
	Maximum Depth:	100	Degree:	13	Solver:	Lbfgs
	Minimum Sample Split:	10	Probability:	True	Random state:	None
	Minimum Sample Leaf:	5	Class weight:	Balanced	Class weight:	Balanced
	Maximum Features:	Log2			Verbose:	1
	Bootstrap:	True				
	Maximum Samples:	0.7				
	Cost Complexity Pruning Alpha:	0.05				
	Class-weight:	Balanced				

Number of trees = number of possible decisions; Random State = seed value that ensures reproducibility by controlling the randomness in algorithms; Maximum Depth = longest path from input to solution in a decision tree; Minimum Sample Split = minimum number of samples required to split an internal node in a decision tree; Minimum Sample Leaf = minimum number of samples required to be present in a leaf node; Maximum Features = maximum number of representative features for a decision tree to generate an output; Bootstrap = technique where individual trees are trained on randomly sampled subsets of data; Maximum Samples = maximum number of samples to use when training each tree in a bootstrap aggregation method; Cost Complexity Pruning Alpha = parameter that simplifies the structure of the decision tree; Class-weight = classification model that assigns different importance to different classes when some are imbalanced; Regularization = technique used to prevent overfitting by penalizing large coefficients in the model; Kernel = function that analyzes patterns, converting them into a linear variable; Poly = polynomial; Degree = highest number of the polynomial function’s power; Maximum Number of Iterations = the maximum number of times an algorithm will attempt to optimize before stopping; Solver = optimization algorithm that finds the best parameters for a model by minimizing the loss of function; Liblinear = library for large linear classification; Lbfgs = limited-memory Broyden–Fletcher–Goldfarb–Shanno; Verbose = function that controls how detailed the output of the algorithm will be.

**Table 2:** General characteristics of the study cohort. Quantitative data are given as mean [range]. Qualitative data are given as numbers and percentages.

Variable	Value Median [Range] or Numbers (Percentages)
Age (years)	65 [61–69]
Digital rectal examination	
Positive, n (%)	94 (61.03%)
Negative, n (%)	60 (38.96%)
PSA value (ng/mL)	10.27 [3.5–70.0]
PSA density (ng/mL^2^)	0.209 [0.012–1.24]
Prostatic volume (cm^3^)	47.23 [36.265–60.3]
Prostatic nodules	201
PI-RADS Score, n (%)	
3	37 (18.4%)
4	107 (53.23%)
5	57 (28.35%)
Lesion maximum diameter (mm)	14.65 [8–32]
Nodule location, n (%)	
Right side	97 (48.25%)
Left side	88 (43.78%)
Extending into both lobes	16 (7.96%)
Targeted cores per lesion	3 [3,4]
ISUP Grade per nodule, n (%)	
ISUP 1	78 (38.8%)
ISUP 2	87 (43.28%)
ISUP 3	31 (15.42%)
ISUP 4	3 (1.49%)
ISUP 5	2 (0.99%)

**Table 3:** Performance in the radiomic parameters of the three classification models for differentiating ISUP 1 versus ISUP 2–5 lesions, filtered according to the three correlation thresholds.

		TPR	TNR	PPV	NPV	FPR	FNR	FDR	Accuracy
Logistic Regression	No correlation	0.8125	0.3200	0.6047	0.5714	0.6800	0.1875	0.3953	0.5965
	Partial correlation	0.9375	0.0800	0.5660	0.5000	0.9200	0.0625	0.4340	0.5088
	Full correlation	0.9375	0.0800	0.5660	0.5000	0.9200	0.0625	0.4340	0.5614
Support Vector Machine	No correlation	0.5938	0.5200	0.6129	0.5000	0.4800	0.4063	0.3871	0.5569
	Partial correlation	0.4063	0.7200	0.6500	0.4865	0.2800	0.5938	0.3500	0.563
	Full correlation	0.2500	0.9200	0.8000	0.4894	0.0800	0.7500	0.2000	0.5850
Random Forest	No correlation	0.6563	0.8000	0.8077	0.6452	0.2000	0.3438	0.1923	0.7281
	Partial correlation	0.9375	0.8250	0.8428	0.9297	0.1750	0.0625	0.1923	0.8813
	Full correlation	0.5625	0.7200	0.7200	0.5625	0.2800	0.4375	0.2800	0.6412

TPR = true positive rate; TNR = true negative rate; PPV = positive predictive value; NPV = negative predictive value; FPR = false positive rate; FNR = false negative rate; FDR = false discovery rate.

**Table 4:** Performance in the combined clinical and radiomic evaluation of the three classification models for differentiating ISUP 1 versus ISUP 2–5 lesions, filtered according to the three correlation thresholds.

		TPR	TNR	PPV	NPV	FPR	FNR	FDR	Accuracy
Logistic Regression	No correlation	0.9583	0.1355	0.5750	0.7272	0.8644	0.0416	0.4250	0.5877
	Partial correlation	0.9722	0.0677	0.5600	0.6666	0.9322	0.0277	0.4400	0.5648
	Full correlation	0.9722	0.0847	0.5654	0.7142	0.9152	0.0277	0.4354	0.5725
Support Vector Machine	No correlation	0.7024	0.5000	0.5842	0.6269	0.2500	0.1488	0.4158	0.6012
	Partial correlation	0.7638	0.8644	0.8744	0.7500	0.1355	0.2361	0.1269	0.8091
	Full correlation	0.4722	1.000	1.000	0.6082	-	0.5277	-	0.7099
Random Forest	No correlation	0.7777	0.8983	0.9032	0.7611	0.1016	0.2222	0.0967	0.8320
	Partial correlation	0.9414	0.8808	0.8875	0.9375	0.1191	0.0585	0.1125	0.9111
	Full correlation	0.7361	0.8305	0.8412	0.7205	0.1694	0.2638	0.1587	0.7786

TPR = true positive rate; TNR = true negative rate; PPV = positive predictive value; NPV = negative predictive value; FPR = false positive rate; FNR = false negative rate; FDR = false discovery rate.

**Table 5:** Performance in radiomic parameters of the three classification models for differentiating between ISUP 2 and 3, filtered according to the three correlation thresholds.

		TPR	TNR	PPV	NPV	FPR	FNR	FDR	Accuracy
Logistic Regression	No correlation	0.4667	0.5200	0.3684	0.6190	0.4800	0.5333	0.6316	0.4934
	Partial correlation	0.3333	0.7200	0.4167	0.6429	0.2800	0.6667	0.5833	0.5266
	Full correlation	0.3333	0.8000	0.5000	0.6667	0.2000	0.6667	0.5000	0.5666
Support Vector Machine	No correlation	0.2000	0.8800	0.5000	0.6471	0.1200	0.8000	0.5000	0.6250
	Partial correlation	0.5333	0.8400	0.6667	0.7500	0.1600	0.4667	0.3333	0.6866
	Full correlation	0.0667	1.0000	1.0000	0.6410	-	0.9333	-	0.5333
Random Forest	No correlation	0.6667	0.7200	0.5882	0.7826	0.2800	0.3333	0.4118	0.6934
	Partial correlation	0.7333	0.8800	0.7857	0.8462	0.1200	0.2667	0.2143	0.8250
	Full correlation	0.7333	0.7600	0.6471	0.8261	0.2400	0.2667	0.3529	0.7467

TPR = true positive rate; TNR = true negative rate; PPV = positive predictive value; NPV = negative predictive value; FPR = false positive rate; FNR = false negative rate; FDR = false discovery rate.

**Table 6:** Performance in the combined clinical and radiomic evaluation of the three classification models for differentiating between ISUP 2 and 3, filtered according to the three correlation thresholds.

		TPR	TNR	PPV	NPV	FPR	FNR	FDR	Accuracy
Logistic Regression	No correlation	0.5128	0.6481	0.5931	0.5711	0.3519	0.4872	0.4596	0.5805
	Partial correlation	0.5128	0.7962	0.6451	0.6935	0.2037	0.4871	0.3548	0.6545
	Full correlation	0.4358	0.8148	0.6296	0.6667	0.1851	0.5641	0.3703	0.6253
Support Vector Machine	No correlation	0.4358	0.9814	0.9444	0.7066	0.0185	0.5641	0.0555	0.7087
	Partial correlation	0.6103	0.8667	0.8207	0.6898	0.1333	0.3897	0.1785	0.7385
	Full correlation	0.2307	1.000	1.000	0.6428	-	0.7692	-	0.6774
Random Forest	No correlation	0.8974	0.8888	0.8536	0.9230	0.1111	0.1025	0.1463	0.8924
	Partial correlation	0.9230	0.9074	0.8780	0.9423	0.0925	0.0769	0.1219	0.9139
	Full correlation	0.7986	0.8563	0.8475	0.8096	0.1436	0.201	0.1524	0.8275

TPR = true positive rate; TNR = true negative rate; PPV = positive predictive value; NPV = negative predictive value; FPR = false positive rate; FNR = false negative rate; FDR = false discovery rate.

## Data Availability

The original contributions presented in this study are included in the article. Further inquiries can be directed to the corresponding author.

## Supplementary Materials

The following supporting information can be downloaded at: https://www.mdpi.com/article/10.3390/cancers17122035/s1, Figure S1: Sample cases classified in the testing phase of the ISUP 1 versus ISUP 2–5 experiment; Figure S2: Sample cases classified in the testing phase of the ISUP 2 versus ISUP 3 experiment; Table S1: Textural features used in the No correlation and Partial correlation settings; Table S2: Performance in the clinical parameters of the three classification models for differentiating ISUP 1 versus ISUP 2–5 lesions, filtered according to the three correlation thresholds; Table S3: Performance in clinical parameters for the three classification models for differentiating between ISUP 2 and 3, filtered according to the three correlation thresholds.
