# Supplementary material for: ISUP Grade Prediction of Prostate Nodules on T2WI Acquisitions Using Clinical Features, Textural Parameters and Machine Learning-Based Algorithms
Source: Cancers (Basel). 2025 Jun 18;17(12):2035. doi: 10.3390/cancers17122035 (PMC12190648; doi:10.3390/cancers17122035)
Supplement: Supplementary file 1 [file cancers-17-02035-s001.zip › cancers-3624948-supplementary.pdf]

No Correlation Features			Partial Correlation Features
**Shape_LeastAxis Lenght**	**GLCM_lmc1**	GLRLM_LongRunHighGrayLevelEmphasis	Shape_Elongation
Shape_Sphericity	GLCM_lmc2	GLRLM_LowGray LevelRunEmphasis	Shape_Flatness
Shape_Surface VolumeRatio	GLCM_JointAverage	GLRLM_RunEntropy	Shape_LeastAxis Lenght
Firstorder_10 Percentile	GLCM_JointEnergy	GLRLM_RunLenght NonUniformity	Shape_Sphericity
Firstorder_90 Percentile	GLCM_JointEntropy	GLRLM_RunLenght NonUniformity Normalized	Shape_Surface VolumeRatio
Firstorder_Entropy	GLCM_MCC	GLRLM_Run Percentage	Firstorder_10 Percentile
Firstorder_ InterquartileRange	GLCM_Maximum Probability	GLRLM_RunVariance	Firstorder_Entropy
Firstorder_Kurtosis	GLCM_Sum Average	GLRLM_ShortRun Emphasis	Firstorder_Kurtosis
Firstorder_Maximum	GLCM_Inverse Variance	GLRLM_ShortRun HighGrayLevel Emphasis	Firstorder_Maximum
Firstorder_Mean AbsoluteDeviation	GLCM_SumEntropy	Diagnostics_Label	Firstorder_Skewness
Firstorder_Mean	GLCM_SumSquares	GLSZM_GrayLevel NonUniformity	Firstorder_Uniformity
Firstorder_Median	GLDM_Dependence Entropy	GLSZM_Graylevel NonUniformity Normalized	GLCM_Auto Correlation
Firstorder_Minimum	GLDM_Dependence NonUniformity	GLSZM_GrayLevel Variance	GLCM_ClusterShade
Firstorder_Robust MeanAbsolute Deviation	GLDM_Dependence NonUniformity Normalized	GLSZM_HighGray LevelZoneEmphasis	GLCM_Contrast
Firstorder_RootMean Squared	GLDM_Dependence Variance	GLSZM_LargeArea Emphasis	GLCM_Correlation
Firstorder_Skewness	GLDM_GrayLevel NonUniformity	GLSZM_LargeArea HighGrayLevel Emphasis	GLCM_ld
Firstorder_Uniformity	GLDM_GrayLevel Variance	GLSZM_LargeArea LowGrayLevel Emphasis	GLCM_ldmn
Firstorder_Variance	GLDM_HighGrayLevelEmphasis	GLSZM_LowGray LevelZoneEmphasis	GLCM_lmc1
GLCM_Auto Correlation	GLDM_Large Dependence Emphasis	GLSZM_SizeZone NonUniformity	GLDM_Dependence NonUniformity Normalized
GLCM_Cluster Prominence	GLDM_Large DependenceHigh GrayLevelEmphasis	GLSZM_SizeZone NonUniformity Normalized	GLDM_Dependence Variance

GLCM_ClusterShade	GLDM_Large DependenceLowGray LevelEmphasis	GLSZM_SmallArea Emphasis	GLDM_Large DependenceLowGray LevelEmphasis
GLCM_Cluster Tendency	GLDM_LowGrayLev elEmphasis	GLSZM_SmallArea HighGrayLevel Emphasis	GLDM_Small DependenceLowGray LevelEmphasis
GLCM_Contrast	GLDM_Small Dependence Emphasis	GLSZM_SmallArea LowGrayLevel Emphasis	Diagnostics_Label
GLCM_Correlation	GLDM_Small DependenceHigh GrayLevelEmphasis	GLSZM_ZoneEntrop y	GLSZM_SizeZone NonUniformity Normalized
GLCM_Difference Average	GLDM_Small DependenceLowGray LevelEmphasis	GLSZM_Zone Percentage	GLSZM_SmallArea LowGrayLevel Emphasis
GLCM_Difference Entropy	GLRLM_GrayLevel NonUniformity	GLSZM_ZoneVarianc e	NGTDM_Coarseness
GLCM_Difference Variance	GLRLM_GrayLevel NonUniformity Normalized	NGTDM_Busyness	NGTDM_Contrast
GLCM_ld	GLRLM_GrayLevel Variance	NGTDM_Coarseness	NGTDM_Strenght
GLCM_ldm	GLRLM_HighGray LevelRunEmphasis	NGTDM_Complexity	
GLCM_ldmn	GLRLM_LongRun Emphasis	NGTDM_Contrast	
GLCM_ldn	GLRLM_LongRunHi ghGrayLevelEmphasi s	NGTDM_Strenght	

Supplementary Table 1. Textural features used in the No correlation and Partial correlation settings. GLDM = Gray level dependence matrix; GLCM = Gray level co-occurrence matrix; GLRLM = Gray level run length matrix; GLSZM = Gray level size zone matrix; NGTDM = neighborhood gray-tone difference matrix; Id = Inverse difference; Idn = Inverse difference normalized; Idm = Inverse difference moment; Idmn = Inverse difference moment normalized; Imc1 = Informational measure of correlation 1; Imc2 = Informational measure of correlation 2.

		TPR	TNR	PPV	NPV	FPR	FNR	FDR	Accuracy
**Logistic Regression**	**No correlation**	0.4871	0.4444	0.6551	0.2857	0.5555	0.5128	0.3448	0.4736
	**Partial correlation**	0.5641	0.6666	0.7857	0.4137	0.3333	0.4358	0.2142	0.5964
	**Full correlation**	0.2820	0.7777	0.7333	0.3333	0.2222	0.7179	0.2666	0.4385
**Support Vector Machine**	**No correlation**	0.5384	0.6666	0.7777	0.4000	0.3333	0.4615	0.2222	0.5789
	**Partial correlation**	0.6153	0.6111	0.7741	0.4230	0.3888	0.3846	0.2258	0.6140
	**Full correlation**	0.4871	0.6666	0.7600	0.3750	0.3333	0.5128	0.2400	0.5438
**Random Forest**	**No correlation**	0.8974	0.055	0.6730	0.2000	0.9444	0.1025	0.3269	0.6315
	**Partial correlation**	0.9230	0.1111	0.6923	0.4000	0.8888	0.0769	0.3076	0.6666
	**Full correlation**	0.8158	0.1589	0.4924	0.4631	0.8410	0.1841	0.5076	0.4874

Supplementary Table 2. Performance in the clinical parameters of the three classification models for differentiating ISUP 1 versus ISUP 2 – 5 lesions, filtered according to the three correlation thresholds.
TPR = true positive rate; TNR = true negative rate; PPV = positive predictive value; NPV = negative predictive value; FPR = false positive rate; FNR = false negative rate; FDR = false discovery rate.

		TPR	TNR	PPV	NPV	FPR	FNR	FDR	Accuracy
Logistic Regression	No correlation	0.4656	0.6768	0.5903	0.5588	0.3232	0.5344	0.4097	0.5712
	Partial correlation	0.5333	0.6800	0.5000	0.7083	0.3200	0.4666	0.5000	0.6250
	Full correlation	0.5333	0.5600	0.4210	0.6666	0.4400	0.4666	0.5789	0.5500
Support Vector Machine	No correlation	0.600	0.6800	0.5294	0.7391	0.3200	0.4000	0.4705	0.6500
	Partial correlation	0.4666	0.8400	0.6363	0.7241	0.1600	0.5333	0.3636	0.7009
	Full correlation	0.4857	0.7389	0.6504	0.5896	0.2611	0.5143	0.3496	0.6123
Random Forest	No correlation	0.6109	0.7200	0.5625	0.7500	0.2800	0.3891	0.4375	0.6750
	Partial correlation	0.8000	0.7609	0.6666	0.8363	0.2391	0.2000	0.3333	0.7750
	Full correlation	0.6265	0.6900	0.6690	0.6488	0.3099	0.3734	0.3309	0.6583

Supplementary Table 3. Performance in clinical parameters for the three classification models for differentiating between ISUP 2 and 3, filtered according to the three correlation thresholds.
TPR = true positive rate; TNR = true negative rate; PPV = positive predictive value; NPV = negative predictive value; FPR = false positive rate; FNR = false negative rate; FDR = false discovery rate.

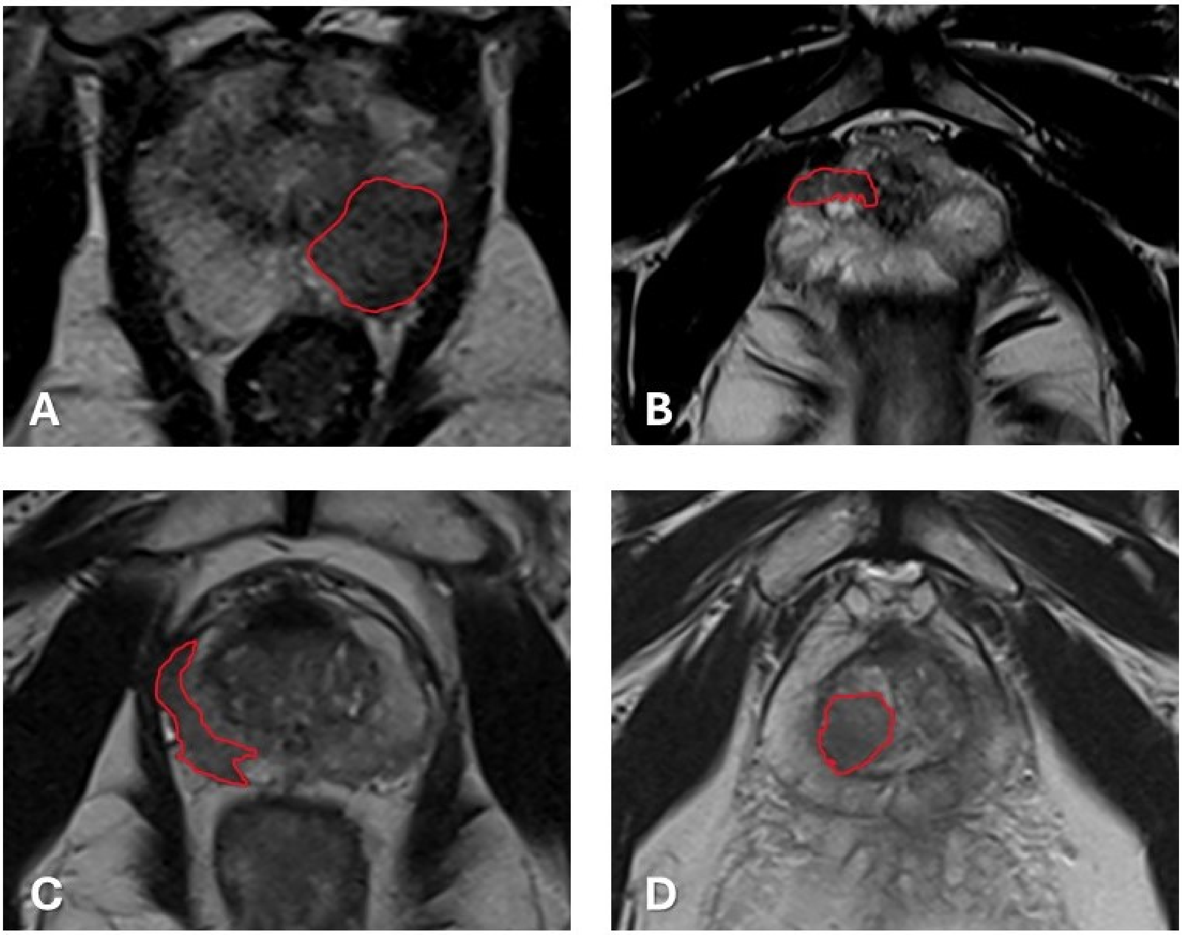

Supplementary Figure 1. Sample cases classified in the testing phase of the ISUP 1 versus ISUP 2 – 5 experiment. **A.** True positive case: lesion in the peripheral zone of the left prostatic lobe, at the base of the gland, 21 mm, PI-RADS 5, ISUP 3, classified correctly. **B.** True negative case: lesion in the transitional zone of the right prostatic lobe, at the apex, 13 mm, PI-RADS 4, ISUP 1, classified correctly. **C.** False positive case: lesion in the peripheral zone of the right prostatic lobe, middle segment, 18 mm, PI-RADS 3, ISUP 1, misclassified as ISUP 2 – 5. **D.** False negative case: lesion in the transitional zone of the right prostatic lobe, middle segment, 14 mm, PI-RADS 4, ISUP 3, misclassified as ISUP 1.

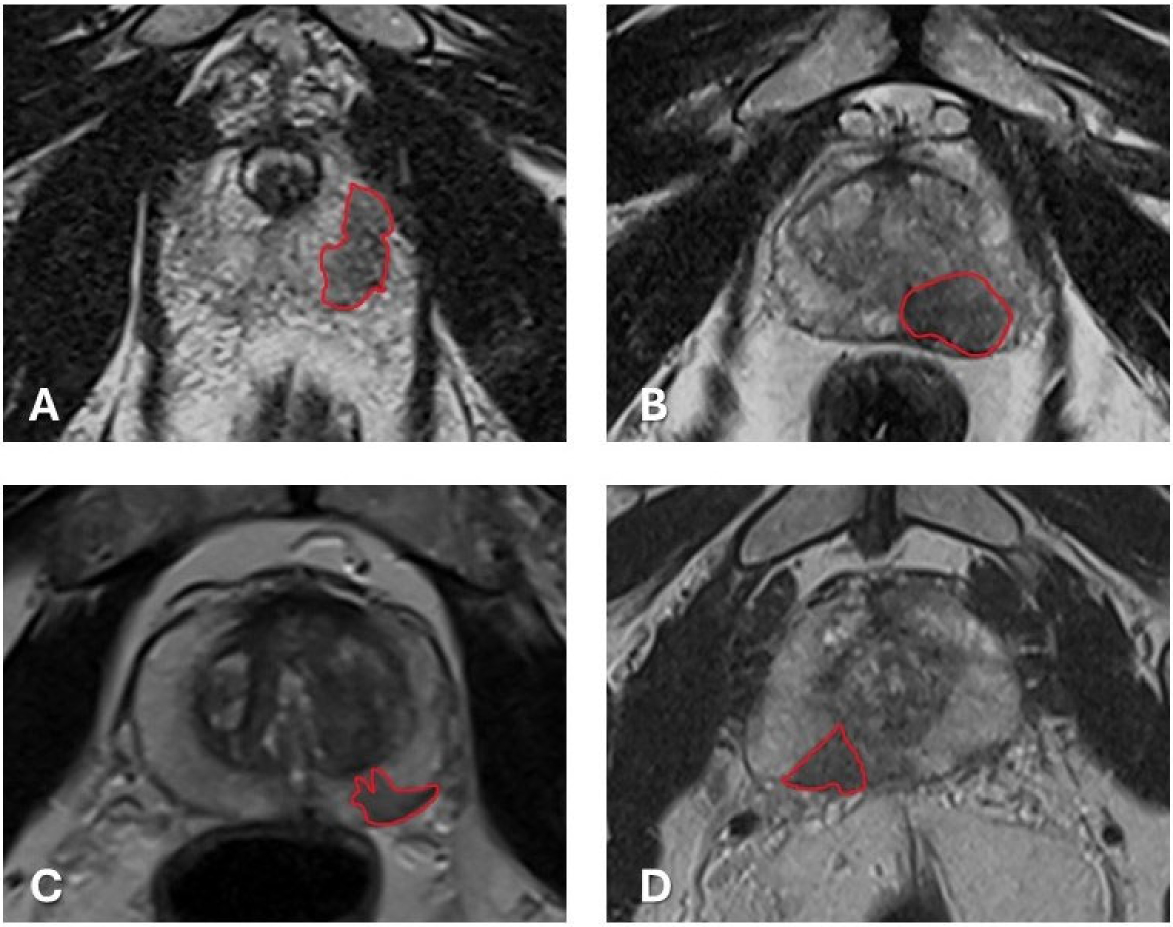

Supplementary Figure 2. Sample cases classified in the testing phase of the ISUP 2 versus ISUP 3 experiment. **A.** True positive case: lesion in the peripheral zone of the left prostatic lobe, at the apex of the gland, 12 mm, PI-RADS 4, ISUP 3, classified correctly. **B.** True negative case: lesion in the peripheral zone of the left prostatic lobe, middle segment, 13 mm, PI-RADS 4, ISUP 2, classified correctly. **C.** False positive case: lesion in the peripheral zone of the left prostatic lobe, at the base of the gland, 13 mm, PI-RADS 4, ISUP 2, misclassified as ISUP 3. **D.** False negative case: lesion in the peripheral zone of the right prostatic lobe, middle segment, 14 mm, PI-RADS 4, ISUP 3, misclassified as ISUP 2.
